# Immunoglobulin replacement products protect against SARS-CoV-2 infection in vivo despite poor neutralizing activity

**DOI:** 10.1172/jci.insight.176359

**Published:** 2024-02-08

**Authors:** Ofer Zimmerman, Alexa Michelle Altman Doss, Baoling Ying, Chieh-Yu Liang, Samantha R. Mackin, Hannah G. Davis-Adams, Lucas J. Adams, Laura A. VanBlargan, Rita E. Chen, Suzanne M. Scheaffer, Pritesh Desai, Saravanan Raju, Tarisa L. Mantia, Caitlin C. O’Shaughnessy, Jennifer Marie Monroy, H. James Wedner, Christopher J. Rigell, Andrew L. Kau, Tiffany Biason Dy, Zhen Ren, Jackson S. Turner, Jane A. O’Halloran, Rachel M. Presti, Peggy L. Kendall, Daved H. Fremont, Ali H. Ellebedy, Michael S. Diamond

**Affiliations:** 1 Department of Medicine, and; 2 Department of Pediatrics, Washington University in St. Louis, St. Louis, Missouri, USA.; 3 Department of Pathology and Immunology,; 4 Department of Molecular Microbiology,; 5 Center for Women’s Infectious Disease Research,; 6 The Andrew M. and Jane M. Bursky Center for Human Immunology & Immunotherapy Programs, and; 7 Center for Vaccines and Immunity to Microbial Pathogens, Washington University School of Medicine, St. Louis, Missouri, USA.

**Keywords:** COVID-19, Immunology, Immunoglobulins

## Abstract

Immunoglobulin (IG) replacement products are used routinely in patients with immune deficiency and other immune dysregulation disorders who have poor responses to vaccination and require passive immunity conferred by commercial antibody products. The binding, neutralizing, and protective activity of intravenously administered IG against SARS-CoV-2 emerging variants remains unknown. Here, we tested 198 different IG products manufactured from December 2019 to August 2022. We show that prepandemic IG had no appreciable cross-reactivity or neutralizing activity against SARS-CoV-2. Anti-spike antibody titers and neutralizing activity against SARS-CoV-2 WA1/2020 D614G increased gradually after the pandemic started and reached levels comparable to vaccinated healthy donors 18 months after the diagnosis of the first COVID-19 case in the United States in January 2020. The average time between production to infusion of IG products was 8 months, which resulted in poor neutralization of the variant strain circulating at the time of infusion. Despite limited neutralizing activity, IG prophylaxis with clinically relevant dosing protected susceptible K18-hACE2–transgenic mice against clinical disease, lung infection, and lung inflammation caused by the XBB.1.5 Omicron variant. Moreover, following IG prophylaxis, levels of XBB.1.5 infection in the lung were higher in FcγR-KO mice than in WT mice. Thus, IG replacement products with poor neutralizing activity against evolving SARS-CoV-2 variants likely confer protection to patients with immune deficiency disorders through Fc effector function mechanisms.

## Abstract 2

<p>Immunoglobulin replacement products that are used as therapy in patients with immunodeficiency disorders confer in vivo protection against SARS-CoV-2 variant XBB.1.5 despite poor neutralization activity.</p>

## Graphical abstract

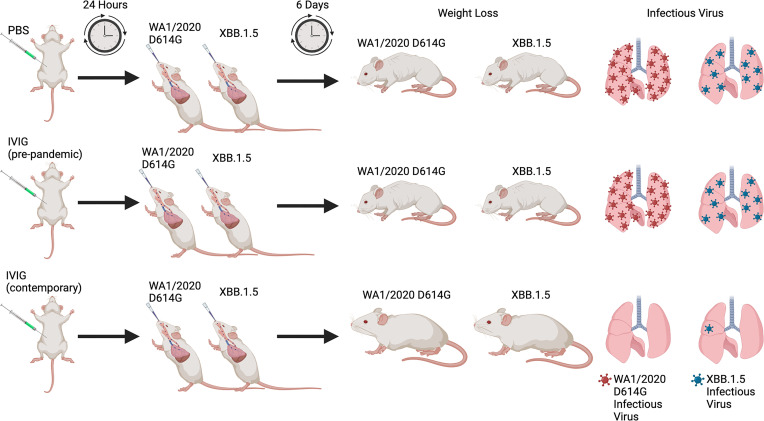

## Introduction

Immunoglobulin (IG) replacement therapy is widely used in patients with primary and secondary immune deficiency syndromes to protect against infections (1). Primary immune deficiency syndromes that require treatment with IG replacement include primary antibody deficiency disorders (e.g., common variable immune deficiency disorder, specific antibody deficiency, and primary hypogammaglobulinemia) and combined immune deficiency disorders (e.g., severe combined immune deficiency). Secondary immune deficiency syndromes benefiting from IG replacement include hypogammaglobulinemia after anti-CD20 monoclonal antibody (mAb) therapy (e.g., rituximab or ocrelizumab) and hematologic malignancies (1). In addition, there are many other conditions that require individuals to be treated with IG replacement therapy: neuroimmunologic disorders (e.g., chronic inflammatory demyelinating polyneuropathy, multifocal motor neuropathy, Guillain-Barré syndrome, and myasthenia gravis), autoimmunity (e.g., immune thrombocytopenia, autoimmune hemolytic anemia, and Kawasaki disease), alloimmune conditions (e.g., hemolytic disease of the fetus and newborn, posttransfusion purpura, and antibody-mediated organ transplant rejection), and infections (e.g., toxic shock syndrome) (1). Patients with primary and secondary immunodeficiency disorders treated with subcutaneous (s.c.) or intravenous (i.v.) IG products (SCIG and IVIG, respectively) are dependent on the passive humoral immunity conferred by their weekly or monthly infusions, respectively.

There are at least 15 different commercially available IG products in the United States (1). Production of IG replacement products takes up to 1 year from sample donation to distribution (2, 3). Each vial contains IG (more than 95% IgG, with trace amounts of IgA or IgM) pooled from plasma of thousands of donors (1, 3), with each manufacturer recruiting their own donors within the United States. Since the emergence of the SARS-CoV-2 pandemic in late 2019, there has been uncertainty as to whether IG replacement products confer protection against infection by emerging variants of concern. Data regarding anti–SARS-CoV-2 antibody levels in IG replacement products collected and manufactured before the pandemic also have been conflicting. One study found that 69% of analyzed prepandemic IG products tested positive for cross-reactive antibodies that bound SARS-CoV-2 spike protein (4), whereas others showed that prepandemic products had no detectable anti–SARS-CoV-2 spike antibodies or neutralizing activity (5–8). Grifols, an IVIG and SCIG manufacturer, first detected anti–SARS-CoV-2 spike antibodies in plasma pools collected in the United States in July 2020 (9). By mid-September 2020, most tested plasma pools had anti–SARS-CoV-2 spike antibodies with increasing titers (9). Takeda, another IG manufacturer, detected SARS-CoV-2 neutralizing antibodies in 12 IVIG and SCIG products collected in March 2020 and released to market in September 2020 (10). IG replacement products have been evaluated for COVID-19 variant neutralization. While anti–Wuhan-1 neutralizing activity gradually increased in tested products over time (10), anti–Omicron BA.1 neutralizing activity was at least 16-fold lower in products released to the market in April 2022 (11). One study showed increases in both anti-spike antibody titer and neutralization activity against WA1/2020 in 10 lots of Hizentra (CSL Behring), with expiration dates beginning December 2022 and ending in December 2023 (12). Another study showed that IG products that efficiently blocked WA1/2020 D614G spike binding to the SARS-CoV-2 entry receptor angiotensin-converting enzyme 2 (ACE2) had poor inhibitory activity against binding of Omicron BA.1 spike to ACE2 (8). Poor neutralizing activity of IG products against Omicron strains BA.1 and BA.1.1 was also observed in another study (13). At present, there are no data to our knowledge on the neutralizing activity of IVIG and SCIG against the more recently emerged XBB.1.5 strain or in vivo protective activity of currently available IG replacement products against circulating SARS-CoV-2 variants. Moreover, while data exist regarding the efficacy of other antibody treatment modalities (mAbs and convalescent plasma) against SARS-CoV-2 infection in mouse models (14, 15), the efficacy of IG replacement products in preclinical animal models is not known and could inform patient care.

Here, we monitored anti–SARS-CoV-2 spike binding and neutralizing antibody titers against multiple SARS-CoV-2 strains (WA1/2020 D614G, Delta, Omicron BA.1, BQ.1.1, and XBB.1.5) in 198 lots of IVIG and SCIG products from 6 different manufacturers administered to patients with antibody deficiency disorders at Washington University School of Medicine (WUSM)/Barnes Jewish Hospital (BJH) from August 2021 to November 2022. These lots were manufactured in the United States from December 2019 to August 2022. We also tested the ability of prepandemic and recently manufactured IG replacement products to protect K18-hACE2–transgenic mice against infection with SARS-CoV-2 WA1/2020 D614G or the XBB.1.5 variant.

## Results

We tested anti–Wuhan-1 spike titers by ELISA in 198 lots of IVIG and SCIG products collected from patients treated at WUSM/BJH (Supplemental Table 1; supplemental material available online with this article; https://doi.org/10.1172/jci.insight.176359DS1). All tested IG products were diluted to an IgG concentration of 1,000 mg/dL (10 mg/mL), which is similar to the optimal serum trough level (960 mg/dL) that is used to protect patients with primary antibody deficiency against pulmonary infection (16) and the mean serum trough IgG level (1,003 ± 221 mg/dL) in our cohort of 27 primary antibody deficiency patients treated with IVIG and SCIG products (Supplemental Figure 1A). This allowed us to test anti–Wuhan-1 spike binding titers in a physiological range and compare them to titers in 20 healthy individuals who had received 2 doses of SARS-CoV-2 mRNA vaccines and had mean serum IgG levels of 1,033 ± 190 mg/dL (Supplemental Figure 1A).

**Figure 1:**
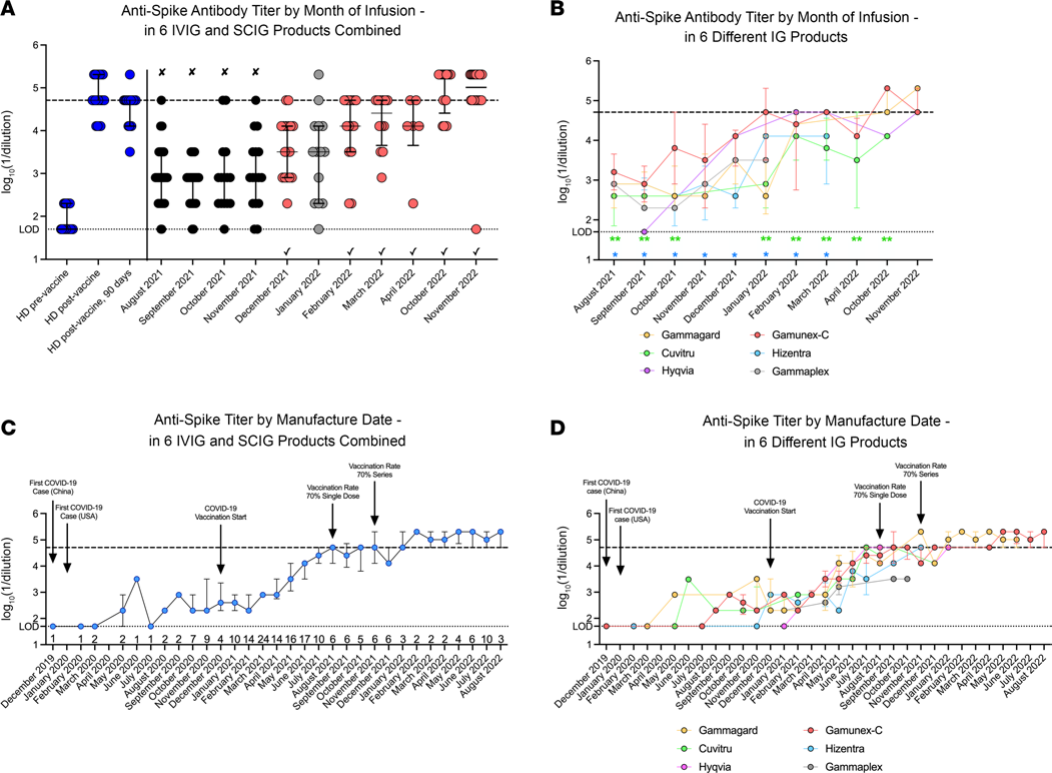
Lag before the detection of anti-spike antibody titers in IG replacement products. (**A**) Anti–Wuhan-1 spike antibody titers were measured in healthy donors (blue dots, *n* = 20) before and 14 and 90 days following completion of 2 doses of COVID-19 mRNA vaccine and in IVIG (*n* = 137) and SCIG (*n* = 61) products infused into patients from August 2021 to November 2022. Black dots (marked with an × above the graph) indicate products with a median anti-spike titer that was not significantly higher than the unvaccinated healthy donor anti-spike titer. Red dots (marked with a √ below the graph) denote products with a median anti-spike titer equivalent to the healthy donor anti-spike titer, 14 days after the second dose of mRNA COVID-19 vaccine. Gray dots indicate products with a median anti-spike titer that was higher than the unvaccinated healthy donor anti-spike titer, but lower than the vaccinated healthy donor anti-spike titer. (**B**) Anti–Wuhan-1 spike antibody titers in 6 different IVIG and SCIG products (Gammagard, orange *n* = 55; Cuvitru, green *n* = 19; Hyqvia, purple *n* = 9; Gamunex-C, red *n* = 75; Hizentra, blue *n* = 33; Gamaplex, gray *n* = 7) infused from August 2021 to November 2022. (**C**) Anti–Wuhan-1 spike antibody titers in 198 lots of IVIG and SCIG products by manufacture date. (**D**) Anti–Wuhan-1 spike antibody titers in 6 different IVIG and SCIG products (Gammagard, orange *n* = 55; Cuvitru, green *n* = 19; Hyqvia, purple *n* = 9; Gamunex-C, red *n* = 75; Hizentra, blue *n* = 33; Gamaplex, gray *n* = 7) by manufacture date. Bars in **A**–**D** indicate median and interquartile range values. LOD, limit of detection (dotted line). Dashed line represents mean anti–Wuhan-1 spike antibody end point titer 14 days following the second dose of SARS-CoV-2 mRNA vaccination in healthy donors (*n* = 20). Numbers above the *x* axis in **C** indicate the number of lots tested in a specific month. *P < 0.05, **P < 0.01 by Kruskal-Wallis with Dunn’s post hoc test (**A**), and mixed effect analysis with Tukey’s posttest correction (**B**). See also Supplemental Figure 1 and Supplemental Table 1.

Until November 2021, IVIG or SCIG products infused in our patients had anti–Wuhan-1 spike antibody titers that were not significantly higher than levels detected in nonvaccinated, COVID-19–naive healthy donors (Figure 1A). In December 2021, anti–Wuhan-1 spike antibody titers in IG products infused in our patients increased and reached the levels of vaccinated healthy donors. However, in January 2022, IG product anti–Wuhan-1 spike titers decreased and were significantly lower than anti–Wuhan-1 spike titers in healthy donors immunized with 2 doses of SARS-CoV-2 mRNA vaccine (Figure 1A). In February 2022, anti–Wuhan-1 spike antibody titers in IG products infused in our patients again reached the levels of vaccinated healthy donors and remained at this level through November 2022 (Figure 1A). Gamunex-C (Grifols), an IVIG product, had higher levels of anti–Wuhan-1 spike antibody titers than 2 of the tested SCIG products, Hizentra (CSL Behring) and Cuvitru (Takeda), when we analyzed data by the month of infusion (Figure 1B).

IVIG and SCIG products manufactured prior to the first surge of COVID-19 cases in the United States in March 2020 had no appreciable anti–Wuhan-1 spike antibodies at the tested concentration of 10 mg/mL (equivalent to human serum level of 1,000 mg/dL) (Figure 1C). Most tested IVIG and SCIG lots manufactured from May 2020 to February 2021 had low titers of anti–Wuhan-1 spike antibodies (Figure 1C). From March 2021 to August 2021, there was a marked increase in anti–Wuhan-1 spike antibody titers in all tested products, with mean titers in IG products reaching levels observed in healthy donors 14 days after administration of a second dose of mRNA vaccine (Figure 1C). This increase started 3 months after the introduction of the first COVID-19 mRNA vaccines in the United States. The level of anti–Wuhan-1 spike antibody titers plateaued after August 2021, when 70% of the adult (18 years and older) US population was vaccinated with at least 1 dose of COVID-19 vaccine (17) and remained stable until January 2022 (Figure 1C). In February 2022, there was a 3-fold increase in the mean anti–Wuhan-1 spike antibody titers in IG products (Figure 1C), which remained stable until August 2022 — the last month tested. Different IVIG and SCIG commercial preparations manufactured in the same month had similar anti–Wuhan-1 spike antibody titers (Figure 1D).

Given the dynamic landscape of SARS-CoV-2 evolution, we examined the temporal relationship between manufacture and infusion dates of each IVIG and SCIG lot tested. On average, patients received IG products manufactured 7.7 months (range 3–25 months) prior to infusion (Supplemental Table 1). Gamunex-C had the shortest time from manufacture to infusion (mean = 6.3 months), compared with Gammagard (mean = 8.8 months, *P* < 0.01) and Cuvitru (mean = 10.4 months, *P* < 0.001) (Supplemental Figure 1B). When comparing IVIG and SCIG as a group, the average time from production to infusion was shorter in IVIG products: 7.3 months versus 8.6 months (*P* = 0.028), respectively (Supplemental Figure 1C).

Together, these data establish that IVIG products manufactured before the pandemic had little cross-reactivity with SARS-CoV-2 Wuhan-1 spike protein, and after the start of the pandemic, antibody titers gradually rose, with the greatest increases beginning at 3 months after the introduction of COVID-19 vaccines. Nonetheless, due to product distribution and turnover, only in December 2021 — almost 2 years after the beginning of the pandemic, did patients in our medical center begin receiving IVIG and SCIG infusions with levels of anti–Wuhan-1 spike antibody comparable to vaccinated healthy individuals. The increase in anti–Wuhan-1 spike antibody titers in products given to our patients was delayed in 2 of the tested SCIG products, likely because of the longer gap between manufacture and infusion dates.

**Figure 2:**
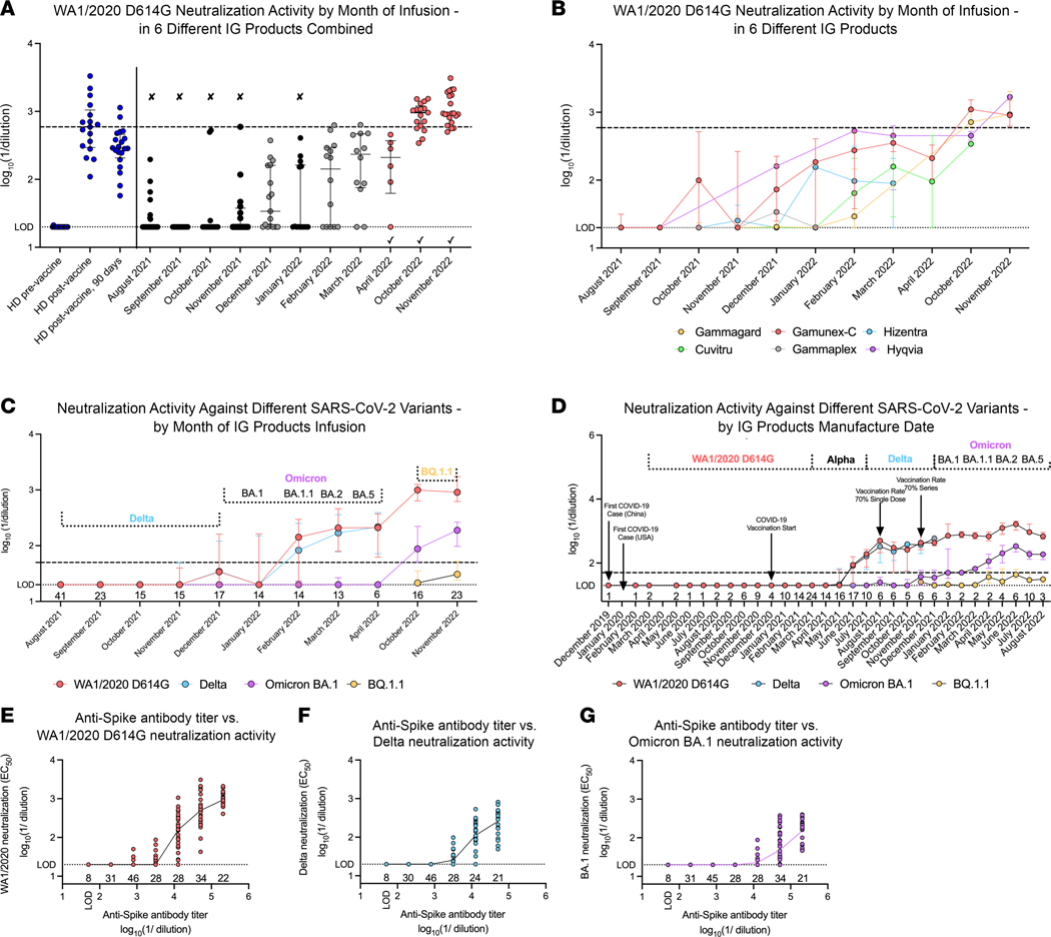
IVIG and SCIG products lack neutralizing activity against the circulating SARS-CoV-2 variant at the time of infusion. (**A**) Neutralization activity in healthy donors (blue dots, *n* = 20) against SARS-CoV-2 WA1/2020 D614G before and 14 and 90 days following completion of 2 doses of COVID-19 mRNA vaccine, and in IVIG (*n* = 136) and SCIG (*n* = 61) products infused into patients from August 2021 to November 2022. Black dots (marked with an × above the graph) indicate products with median anti–WA1/2020 D614G neutralization activity that was not significantly higher than unvaccinated healthy donor serum neutralization activity. Red dots (marked with a √ below the graph) denote products with median anti–WA1/2020 D614G neutralization activity equivalent to healthy donor serum neutralization activity, 14 days after the second dose of mRNA COVID-19 vaccine. Gray dots indicate products with median anti–WA1/2020 D614G neutralization activity that was higher than unvaccinated healthy donor anti–WA1/2020 D614G neutralization activity, but lower than vaccinated healthy donor anti–WA1/2020 D614G neutralization activity. (**B**) Neutralization activity against SARS-CoV-2 WA1/2020 D614G in 6 different IVIG and SCIG products separated by manufacturer (Gammagard, orange *n* = 55; Cuvitru, green *n* = 19; Hyqvia, purple *n* = 9; Gamunex-C, red *n* = 74; Hizentra, blue *n* = 33; Gamaplex, gray *n* = 7) infused from August 2021 to November 2022. (**C**) Neutralizing activity against SARS-CoV-2 WA1/2020 D614G (red dots, *n* = 197), Delta (blue dots, *n* = 157), BA.1 (purple, *n* = 195), and BQ.1.1 (orange, *n* = 38) in IVIG and SCIG products infused from August 2021 to November 2022. (**D**) Neutralizing activity against SARS-CoV-2 WA1/2020 D614G (red dots, *n* = 197), Delta (B.1.617.2; blue dots, *n* = 157), BA.1 (purple, *n* = 195), and BQ.1.1 (orange, *n* = 38) in IVIG and SCIG by manufacture date. (**E**–**G**) Comparison of anti–Wuhan-1 spike antibody titer (*x* axis) and SARS-CoV-2 WA1/2020 D614G (**E**), Delta (**F**), and Omicron BA.1 (**G**) neutralization activity in 157–197 IG products. Bars in **A**–**D** indicate median plus interquartile range values. LOD, limit of detection (dotted line) (**A**–**G**). The dashed line in **A**–**D** represents mean anti–Wuhan-1 neutralizing activity 14 days following the second dose of SARS-CoV-2 mRNA vaccination in healthy donors (*n* = 20) (**A** and **B**) or represents the presumptive protective titer as described in Khoury et al. (19) (**C** and **D**). SARS-CoV-2 variant name above the graph in **C** and **D** indicates the most prevalent circulating strain in the United States during the month in which IVIG/SCIG was infused (**C**) or manufactured (**D**). Numbers above the *x* axis in **C**–**G** indicate the number of lots tested in a specific month (**C** and **D**) or the number of lots with a specific anti–Wuhan-1 spike antibody titer (**E**–**G**). Significance was assessed using Kruskal-Wallis with Dunn’s post hoc test (**A**) or mixed effect analysis with Tukey’s posttest correction (**B**). See also Supplemental Figure 1 and Supplemental Table 1.

We next measured the neutralization activity of the IVIG and SCIG products against ancestral and emerging SARS-CoV-2 strains (Supplemental Table 1). We calculated the half maximal effective concentration (EC_50_) values based on dilution of IVIG and SCIG products after normalization to an initial concentration of 10 mg/mL (equivalent to human serum level of 1,000 mg/dL). Before October 2021, the majority of IVIG and SCIG products infused into our patients had no detectable neutralizing activity against the SARS-CoV-2 WA1/2020 D614G strain (Figure 2, A–C). After December 2021, the mean neutralizing activity of IVIG and SCIG products infused in our patients exceeded the serum neutralizing activity of unvaccinated, COVID-19–naive healthy donors (Figure 2A). Neutralizing activity against the WA1/2020 D614G strain in IG products reached the level of vaccinated healthy controls in April 2022 (Figure 2A). Although different IG products showed some variation in levels of SARS-CoV-2 neutralization of WA1/2020 D614G, these differences did not attain statistical significance (Figure 2B).

We also compared the neutralizing activity of WA1/2020 D614G, Delta (B.1.617.2), Omicron BA.1, and BQ.1.1 by IG replacement products using a longitudinal analysis. Most products had poor activity against the circulating variant at the time of infusion (Figure 2C). No significant difference was observed in neutralizing activity of IG products against the WA1/2020 D614G and Delta strains over many months (Figure 2C). This finding was consistent with results from 2 studies that examined neutralizing activity of the Delta variant by commercial IG products (8, 13) and 1 that followed individuals immunized with mRNA vaccines, with or without history of COVID-19 infection (18). Neutralizing activity against Omicron BA.1 strain was not observed in infused products until April 2022, 4 months after Omicron began circulating in the United States (Figure 2C). Even in October and November of 2022, the mean neutralizing titer against Omicron BA.1 strain was 10-fold lower than against SARS-CoV-2 WA1/2020 D614G. At the time of BQ.1.1 emergence, most infused products had no to poor inhibitory activity against this variant (Figure 2C). Even in products that neutralized BQ.1.1 infection, EC_50_ values were lower than 1/50, a value that has been proposed as a correlate of protection in vaccinated individuals (19).

When examining neutralizing titers by manufacture date, inhibitory activity against SARS-CoV-2 WA1/2020 D614G was not observed in most tested products until May 2021, 17 months after the first COVID-19 case in the United States (Figure 2D and Supplemental Figure 1D). From May to August 2021, in IVIG and SCIG products, there was a marked and consistent increase in neutralizing activity against SARS-CoV-2 WA1/2020 D614G and Delta strains (Figure 2D and Supplemental Figure 1, D and E). IVIG and SCIG products manufactured from August 2021 to August 2022 had a stable level of neutralizing activity against SARS-CoV-2 WA1/2020 D614G, with small month-to-month changes (Figure 2D and Supplemental Figure 1D). Similar findings were observed with the Delta strain (Figure 2D and Supplemental Figure 1E). Whereas IVIG and SCIG manufactured during the initial stages of the pandemic did not neutralize WA1/2020 strains, products manufactured when the Delta strain was circulating could in theory have conferred protection to patients because they had neutralizing activity against the Delta strain (Figure 2D). However, at that time, these products were not available to patients treated in our medical center network, as they were still receiving products manufactured approximately 8 months prior, which had little anti–SARS-CoV-2 (WA1/2020 D614G or Delta) activity.

Low levels of anti–Omicron BA.1 neutralization activity were first observed in products manufactured 2 months prior to its emergence, likely due to cross-reactivity of anti-spike antibodies induced by vaccination and/or infection with ancestral strains or earlier variants (Figure 2D and Supplemental Figure 1F). In March 2022, 4 months after the emergence of Omicron in the United States, neutralizing activity against Omicron BA.1 in IG products increased to levels that have exceeded 1/50 serum titers and should have conferred protection to infused patients (Figure 2D and Supplemental Figure 1F). However, even 6 months after the emergence of Omicron BA.1 in the United States, levels of neutralizing activity against this strain were 5-fold lower than against WA1/2020 D614G. Neutralizing activity against the BQ.1.1 variant was first observed in IG products manufactured in April 2022, although levels remained low through August 2022 (Figure 2D). We assessed correlations between anti–Wuhan-1 spike antibody titer and neutralizing activity against SARS-CoV-2 WA1/2020 D614G, Delta, and Omicron BA.1 in all tested IG products (Figure 2, E–G). In the low and high range of anti–Wuhan-1 spike antibody titers, there was a correlation between anti-spike titers and neutralizing activity — undetectable neutralizing activity in IG lots with undetectable anti–Wuhan-1 spike antibody titers and high neutralizing activity in products with the highest anti-spike end point titers (Figure 2, E–G). However, across a wider range of anti–Wuhan-1 spike antibody titers from different product lots, we observed substantial variability between binding and neutralizing titers.

Overall, these results suggest that IG products have 2 challenges for conferring protection against SARS-CoV-2 variants: (a) there is a gap between the emergence of new variants and the appearance of neutralizing activity against those variants in manufactured lots; and (b) there is a delay in product distribution and clinical use, such that patients often receive IG products that predate the variant and thus lack sufficient neutralizing activity. Our correlation data also suggests that anti-spike antibody titer may not be a reliable measure for SARS-CoV-2–neutralizing activity in IG products.

We next examined whether currently used IG products would protect against SARS-CoV-2 WA1/2020 D614G and XBB.1.5 strains in vivo. Since the IVIG and SCIG products that were in clinical use at the time of the study had poor neutralization activity against Omicron BQ.1.1 (Figure 2, C and D), we did not expect to detect much neutralizing activity against XBB.1.5 in these products. We tested 15 of the most recent lots from 4 different manufacturers and detected IgG binding to XBB.1.5 in all of them (Supplemental Figure 1G and Supplemental Table 1). However, XBB.1.5 neutralization activity was very low or below the level of detection in 14 of the 15 tested products. In one product, the EC_50_ value against XBB.1.5 was 1/56 (Supplemental Figure 1H and Supplemental Table 1). These findings are consistent with data showing that serum from individuals receiving BNT162b2, mRNA-1273, and CoronaVac vaccines maintained binding against Omicron spike protein but lost neutralizing activity and binding to the receptor binding domain (RBD) (20).

**Figure 3:**
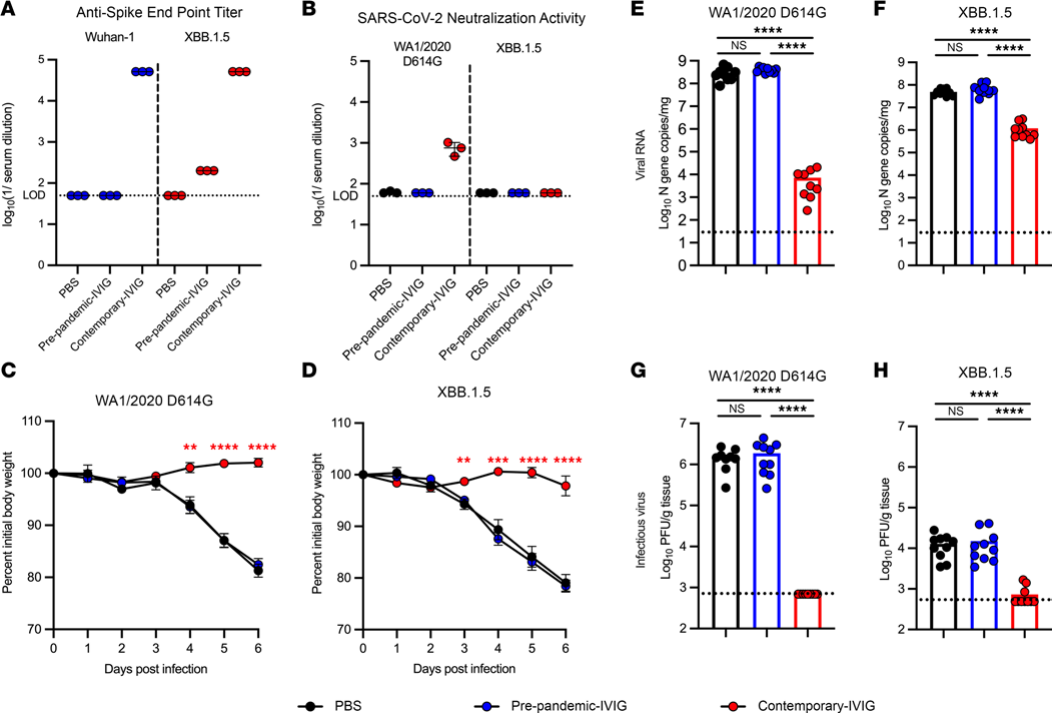
Contemporary IG products protect K18-hAE2–transgenic mice from XBB.1.5 infection despite lacking neutralizing activity. (**A**) Anti–Wuhan-1 or –XBB.1.5 spike human antibody end point titers in naive K18-hACE2–transgenic mice 24 hours after treatment with PBS (black dots, *n* = 3), 600 mg/kg prepandemic IG (blue dots, *n* = 3), or contemporary IG (red dots, *n* = 3). (**B**) Neutralizing activity against SARS-CoV-2 WA1/2020 D614G or XBB.1.5 of serum obtained from naive K18-hACE2–transgenic mice 24 hours after treatment with PBS (black dots, *n* = 3), 600 mg/kg prepandemic IG (blue dots, *n* = 3), or contemporary IG (red dots, *n* = 3). (**C** and **D**) Percentage change in initial body weight in mice treated with PBS (black dots, *n* = 10, 2 independent experiments), prepandemic G (blue dots, *n* = 10), or contemporary IG (red dots, *n* = 10) and challenged with WA1/2020 D614G (**C**) or XBB.1.5 (**D**). (**E**–**H**) Lung SARS-CoV-2 WA1/2020 D614G (**E** and **G**) or XBB.1.5 (**F** and **H**) RNA titers (**E** and **F**) or infectious virus (**G** and **H**) 6 days after infection, in mice treated with PBS (black dots, *n* = 10), prepandemic IG (blue dots, *n* = 10), or contemporary IG (red dots, *n* = 10) 24 hours before intranasal virus challenge. Bars indicate median with interquartile range (**A** and **B**), mean ± SEM (**C** and **D**), or mean (**E**–**H**). **P < 0.01, ***P < 0.001, ****P < 0.0001 by mixed effect analysis with Tukey’s posttest correction (**C** and **D**) or 1-way ANOVA with Tukey’s posttest correction (**E**–**H**).

Recent studies in mice with SARS-CoV-2 or pan-sarbecovirus vaccines have suggested that passively administered immune sera can protect against antigenically shifted coronaviruses, even when neutralizing activity is low, through Fc effector function activity (15, 21, 22). Given these results, and the positive ELISA reactivity against XBB.1.5 of more recent IG products, we assessed their protective efficacy in a mouse model of SARS-CoV-2 infection. Since human and mouse IgG bind to mouse and human Fc γ receptors (FcγRs) with relatively similar binding affinity (23) and since human IVIG is an effective therapy against immune thrombocytopenia in mice because of Fc effector functions (24), we hypothesized that commercial human IG products with low neutralizing activity might still protect mice against SARS-CoV-2 infection. K18-hACE2–transgenic mice, which are susceptible to most SARS-CoV-2 strains (14, 25, 26), were injected via the intraperitoneal (i.p.) route with 600 mg/kg of either prepandemic or currently circulating commercial IVIG products at the time of the experiment, collected from November 2021 to November 2022 (hereafter, contemporary IG), or PBS control. We administered 600 mg/kg of IG because this is the upper limit of a recommended dose for individuals with primary antibody deficiency disorders (1, 27). Twenty-four hours after the injection, we measured anti-spike and neutralizing antibody levels in serum. Mice treated with contemporary IG products had high levels of both anti–Wuhan-1 and –Omicron XBB.1.5 spike antibodies (Figure 3A). Whereas serum neutralizing activity against WA1/2020 D614G was high, neutralizing activity against XBB.1.5 was below the level of detection (Figure 3B). We then challenged K18-hACE2–transgenic mice with either WA1/2020 D614G or XBB.1.5 (intranasal route, 1 × 10^4^ focus-forming units [FFU]) 24 hours after i.p. injection of IG. Whereas substantial (20% to 25%) body weight loss was observed within 6 days of infection with WA1/2020 D614G or XBB.1.5 strains in mice that received PBS or prepandemic IVIG, prophylaxis with contemporary IG products protected against weight loss caused by WA1/2020 D614G and XBB.1.5 infection (Figure 3, C and D). Prophylaxis with contemporary IG product was also associated with a reduction in viral RNA levels in the lungs of mice infected with WA1/2020 D614G (55,000-fold, *P* < 0.0001) and XBB.1.5 (58-fold, *P* < 0.0001), whereas animals given prepandemic IG showed no such protective effect (Figure 3, E and F). We also measured infectious virus levels in mouse lung. Prophylaxis with contemporary IG was associated with a significant decrease in WA1/2020 D614G (2,215-fold, *P* < 0.0001) and XBB.1.5 (18-fold, *P* < 0.0001), whereas animals given prepandemic IG showed no protective effect compared to the PBS control (Figure 3, G and H).

**Figure 4:**
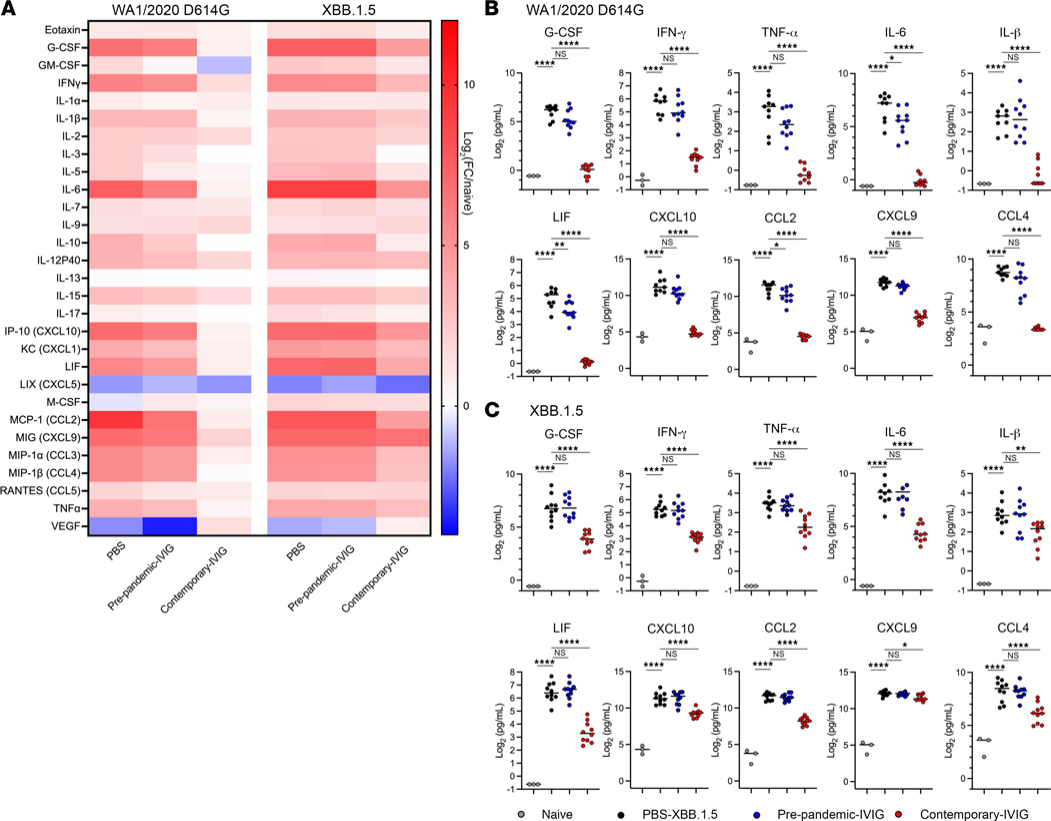
Prophylaxis with contemporary IG was associated with reductions in lung cytokines and chemokines after SARS-CoV-2 challenge of K18-hACE2–transgenic mice. (**A**–**C**) Cytokine and chemokine levels from lung homogenates of K18-hACE2–transgenic mice treated with IG (blue dots, prepandemic, *n* = 10; red dots, contemporary, *n* =10) or PBS (black dots, *n* = 10) and challenged with SARS-CoV-2 WA1/2020 D614G or XBB.1.5. Samples were obtained 6 days after infection. (**A**) For each analyte, fold change was calculated compared to mock-inoculated mice, and log_2_ values were plotted in the color-coded heatmap. (**B** and **C**) Individual cytokine levels were measured in the lung homogenates of WA1/2020 D614G (**B**) or XBB.1.5 (**C**) SARS-CoV-2–infected mice after prophylaxis with prepandemic IG (blue) or contemporary IG (red) or treatment with PBS (black) compared to naive mice (gray). Mean values ± SEM are shown. *P < 0.05, **P < 0.01, ****P < 0.0001 by 1-way ANOVA with Tukey’s posttest correction (**B** and **C**).

Because hyperinflammatory responses contribute to severe COVID-19 and lung disease (28, 29) and as an independent metric of protection, we measured cytokine and chemokine responses in lung homogenates after passive IG transfer and virus challenge (Figure 4, A–C, and Supplemental Figure 2, A and B). Compared with PBS-treated and WA1/2020 D614G–infected animals, mice given contemporary IG and challenged with WA1/2020 D614G showed significant reductions in most cytokines and chemokines in the lungs, almost to the levels seen in naive mice (Figure 4, A and B, and Supplemental Figure 2A). In comparison, prophylaxis with prepandemic IG was associated with small reductions (2- to 3-fold, *P* < 0.05–0.001) in a subset of cytokines (IL-6, LIF, CCL2, GM-CSF, IL-3, IL-5, and IL-10) in mice challenged with WA1/2020 D614G (Figure 4, A and B, and Supplemental Figure 2A), which could be due to antiinflammatory properties of some antibodies (30, 31). In mice given contemporary IG and challenged with XBB.1.5, decreases in most cytokines and chemokines were apparent (Figure 4, A and C, and Supplemental Figure 2B), although the magnitude of protection (2- to 15-fold decrease, *P* < 0.05–0.0001) was less than after WA1/2020 challenge, which correlated with the differences in viral RNA (Figure 3, E and F) and infectious virus levels (Figure 3, G and H). In comparison, in this instance, administration of prepandemic IG had no appreciable protective effect on the levels cytokines or chemokines in mice challenged with XBB.1.5 compared to the PBS control (Figure 4, A and C, and Supplemental Figure 2B).

**Figure 5:**
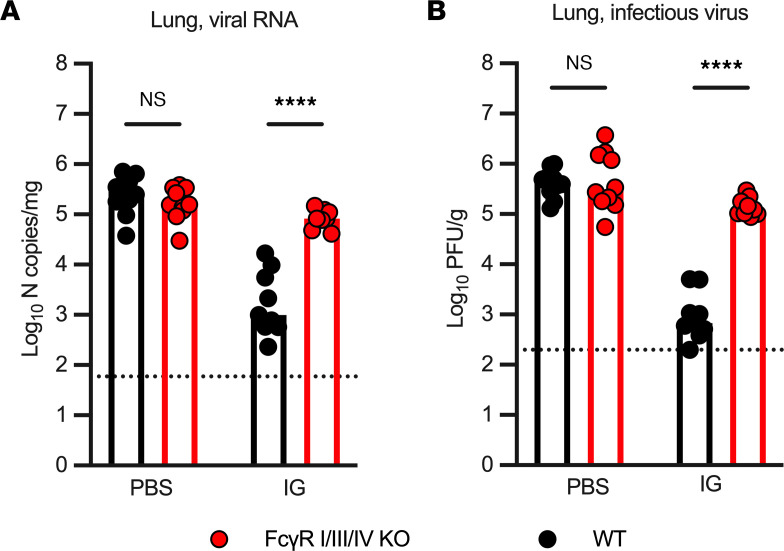
The reduction in XBB.1.5 lung infection following IG prophylaxis is Fc effector function dependent. (**A** and **B**) Levels of XBB.1.5 RNA (**A**) and infectious virus (**B**) in the lungs of C57BL/6J (*n* = 10, black dots) and FcγR I/III/IV–KO mice (*n* = 10, red dots) challenged with SARS-CoV-2 XBB.1.5, 24 hours following administration of PBS or IG prophylaxis. Lungs were collected 2 days after inoculation for virological analysis. Mean values are shown (2 experiments). ****P < 0.0001 by 1-way ANOVA with Tukey’s posttest correction (**A** and **B**).

To better define the mechanism of protection of IG products against the XBB.1.5 variant, we challenged C57BL/6J and FcγR I/III/IV–KO mice with XBB.1.5, 24 hours after i.p. administration of a contemporary IG product. Forty-eight hours later, levels of XBB.1.5 RNA (Figure 5A) and infectious virus (Figure 5B) in the lung were significantly higher (100-fold, *P* < 0. 0001) in FcγR I/III/IV–KO compared with congenic WT mice. In comparison, in the control group treated with PBS prior to virus challenge, there were no differences in XBB.1.5 RNA or infectious virus levels in the lung of WT and FcγR I/III/IV–KO mice (Figure 5, A and B). These findings suggest that IG products with poor XBB.1.5 neutralizing but high spike binding capacity protect against XBB.1.5 infection through Fc effector function activity.

## Discussion

Our findings show that there is a consistent lag between the time that IVIG and SCIG products collected in the United States accumulate high levels of anti–SARS-CoV-2 spike and neutralizing activity against circulating strains and their clinical administration. This delay reflects both the time that passed until anti-spike titers in the population of plasma donors reached levels that are comparable to vaccinated healthy individuals and the long manufacturing process of IG products that includes pooling of plasma collected approximately 9–12 months prior. This lag was further increased by the gap between the manufacture date and the distribution of these products for infusion into patients in infusion centers or at home.

The largest increase in anti–SARS-CoV-2 antibody and neutralizing titers against WA1/2020 D614G and Delta variants in IG products was seen in preparations manufactured 3 months after the emergency use approval of COVID-19 vaccines in December 2020 and plateaued after 70% of the adult population had received at least 1 vaccine dose. These results are consistent with data published by the manufacturer of Gammagard and Hyqvia (32), which showed a rapid increase in the anti-spike–to–anti-nucleocapsid antibody ratio in plasma pools used for IVIG and SCIG manufacture from April 2021 to July 2021. These data suggest that the major source for anti-spike antibodies in the population of plasma donors was SARS-CoV-2 vaccination rather than SARS-CoV-2 infection.

The level of anti-spike binding and neutralizing antibodies against WA1/2020 D614G and Delta remained stable from August 2021 to August 2022, with only small increases in titer. However, neutralizing activity against emerging variants (e.g., Omicron BA.1, BQ.1.1, and XBB.1.5) was relatively poor in products in clinical use. This lag in immunity and production of new IG lots is expected to be an ongoing clinical challenge as new variants emerge (e.g., EG.5.1 and JN.1), even if distribution is optimized.

Since mAbs used for prophylaxis or therapy for COVID-19 infection have lost their neutralizing capacity against contemporary XBB variants (33–37), we tested whether prophylaxis with IG products could confer any level of protection against XBB.1.5. Notably, IG prophylaxis protected against XBB.1.5 infection in K18-hACE2–transgenic mice despite poor neutralization activity. These results are consistent with recent adenovirus-vectored vaccine studies in mice and hamsters, which showed robust protection against XBB.1.5 despite limited neutralizing activity (38). The protection we observed from IG products against weight loss, lung infection, and lung inflammation suggests that non-neutralizing anti-XBB.1.5 antibodies contribute to preventing infection in mice. These results have relevance to studies by other groups and were not a foregone conclusion. Ullah et al. showed that while prophylaxis or therapy with convalescent plasma with moderate to high Fc effector activity delayed mortality and/or improved survival in mice challenged with SARS-CoV-2, convalescent plasma with low Fc effector activity did not (15). Kapolnek et al. found that antibodies from convalescent patients infected with ancestral SARS-CoV-2 strains largely failed to interact with FcRs, despite binding avidly to emerging SARS CoV-2 variants. In contrast, mRNA-1273 vaccine–induced antibodies bound similarly to ancestral and emerging variants and demonstrated relatively equivalent FcR engagement. Since commercial IG products undergo purification processes that could affect human IgG Fc effector function (39, 40), we could not predict whether they would protect against emerging SARS-CoV-2 strains.

While we tested our IG products in vivo using a prophylaxis model, it remains unclear whether IG products can effectively treat acute SARS-CoV-2 infection at a post-exposure stage. Possibly, IG products with good neutralization activity might be effective in a similar way to that of mAbs during the early stages of the pandemic when neutralizing activity against variants was not compromised (41, 42). More studies with animal models and humans and different IG products are required to answer this question.

Our comparative passive transfer studies in congenic WT and FcγR-KO mice suggest that IG product protection against XBB.1.5 was Fc effector function dependent. Recent studies with FcγR-deficient mice and SARS-CoV-2 or other sarbecoviruses also have shown important roles for Fc effector functions in passive and active immunization against antigenically shifted viruses (15, 21, 22, 43). Non-neutralizing antibodies induced by SARS-CoV-2 vaccines have been linked to protection against variant Omicron strains by their ability to engage specific FcγRs and promote clearance (44, 45). As Fc-mediated effector functions in vitro of serum of mRNA-1273– or BNT162b2-vaccinated individuals were not affected by depletion of RBD-specific antibodies (46), antibodies recognizing conserved, non-neutralizing epitopes might contribute to protection against variant strains. Although future studies are required, protection against XBB.1.5 could have been mediated by antibody-dependent cellular cytotoxicity, antibody-dependent cellular phagocytosis, or complement-dependent deposition and phagocytosis or lysis (21, 44–46). The reduction in cytokines observed in both WA1/2020 D614G– and XBB.1.5-challenged mice treated with IG products is likely due to the reduction in viral infection and associated decrease in cell-intrinsic immune cell activation. However, it is possible that IG product engagement of inhibitory FcγRII on various immune cells or type II FcγRs (CD23 and DC-SIGN) also contributed to the reduction in inflammation (47).

We acknowledge several limitations in our study: (a) our studies were derived from samples from a patient cohort at our hospitals and will need to be corroborated with studies at independent sites; (b) we performed challenge studies in K18-hACE2–transgenic and C57BL/6J mice within 1 day of IG administration. Studies that address the durability of protection against heterologous variants are warranted; and (c) extrapolation of findings to even more recently emerging variants (e.g., JN.1) should be performed.

In summary, our data confirm the long delay between the emergence of the COVID-19 pandemic and the time when clinically used IG replacement products accrued a high titer of anti–SARS-CoV-2 spike antibodies. As a result of this production and distribution delay, SARS-CoV-2 neutralization activity of IG products lagged behind the emergence of new variants, a problem that is not easily overcome given the timeline of the collection, production, and distribution of IG products. Nonetheless, despite the poor neutralizing activity against emerging variants, prophylaxis of contemporary IG products in mice effectively limited disease severity and controlled infection and inflammation in the lung after challenge with XBB.1.5, which suggests there likely is some, albeit not optimal, clinical utility for IG products against emerging SARS-CoV-2 variants in patients with primary and secondary antibody deficiency disorders.

## Methods

### IG products collection.

Unused IVIG and SCIG products were collected from patients treated at the Washington University Division of Allergy and Immunology Infusion Center and from patients in the Division of Allergy and Immunology at Washington University. IVIG and SCIG were stored at 4°C. The month of infusion of each lot was documented.

### Healthy donor controls.

Immunocompetent healthy donor volunteer blood samples were obtained as previously described (48).

### Cells.

Vero-TMPRSS2 and Vero-TMPRSS2-hACE2 cells (49) were cultured at 37°C in Dulbecco’s modified Eagle medium (DMEM) supplemented with 10% fetal bovine serum (FBS), 10 mM HEPES pH 7.3, 1 mM sodium pyruvate, 1× nonessential amino acids, 100 U/mL penicillin-streptomycin, and 5 μg/mL blasticidin. Expi293F cells (Thermo Fisher Scientific) were cultured at 37°C in Expi293 expression medium (Thermo Fisher Scientific) on a shaker at 225 RPM.

### Viruses.

The WA1/2020 D614G recombinant strain was obtained from an infectious cDNA clone of the 2019n-CoV/USA_WA1/2020 strain, as described previously (50). The B.1.617.2 Delta isolate was obtained as a gift from R. Webby (St. Jude Children’s Research Hospital, Memphis, Tennessee, USA). The BA.1 (B.1.1.529) isolate (hCoV-19/USA/WI-WSLH-221686/2021) was obtained from an individual in Wisconsin with a nasal swab. The BQ.1.1 and XBB.1.5 isolates were provided by A. Pekosz (Johns Hopkins University, Baltimore, Maryland, USA) and M. Suthar (Emory University, Atlanta, Georgia, USA) as part of the NIH SARS-CoV-2 Assessment of Viral Evolution (SAVE) Program. All viruses were passaged once in Vero-TMPRSS2 cells and subjected to next-generation sequencing after RNA extraction to confirm the introduction and stability of substitutions. All virus experiments were performed in an approved Biosafety level 3 (BSL-3) facility.

### Mice.

Mice were housed in groups and fed standard chow diets. Virus inoculations and sample collections were performed under anesthesia, induced and maintained with ketamine hydrochloride and xylazine. All efforts were made to minimize animal suffering.

Heterozygous K18-hACE2–transgenic mice (strain 2B6.Cg-Tg(K18-ACE2)2Prlmn/J, stock 034860) and C57BL/6J male mice (stock 000664) were obtained from The Jackson Laboratory. FcγR I/III/IV–KO mice (lacking the common γ-chain) were commercially obtained sources (Taconic Biosciences, catalog 583) and then sequentially backcrossed onto a C57BL/6J background (>99%) using Speed Congenics (Charles River Laboratories) and single nucleotide polymorphism analysis (21).

### SARS-CoV-2 spike protein expression.

Genes encoding SARS-CoV-2 Wuhan-1 spike protein (residues 1–1213, GenBank: MN908947.3) and XBB.1.5 (residues 1–1209, GenBank: WHJ03660.1) were cloned into a pCAGGS mammalian expression vector with a C-terminal hexahistidine tag. The spike protein was stabilized in a prefusion form using 6 proline substitutions (F817P, A892P, A899P, A942P, K986P, and V987P) (51), and expression was optimized with a disrupted S1/S2 furin cleavage site and a C-terminal foldon trimerization motif (YIPEAPRDGQAYVRKDGEWVLLSTFL) (52). Expi293F cells were transiently transfected, and proteins were purified by cobalt-affinity chromatography (G-Biosciences) as previously described (53, 54).

### Anti-spike protein ELISA.

Maxisorp ELISA (Thermo Fisher Scientific) plates were coated with SARS-CoV-2 ancestral spike (2 μg/mL) overnight in sodium bicarbonate buffer, pH 9.3. All plates were coated with spike from the same expression and purification batch. Plates were washed 4 times with PBS and 0.05% Tween 20 and blocked with 3% nonfat milk (reconstituted from powder) in PBS/0.05% Tween 20 for 1 hour at 25°C. Plates were then incubated with 50 μL of serially diluted healthy donor samples (eight 4-fold dilutions, starting at 1/50) in 1% nonfat milk/PBS/0.05% Tween 20 for 2 hours at 25°C on a shaker. IG replacement products (IVIG and SCIG) were diluted to 10 mg/mL (average patient and healthy control IgG level) and then treated as described above. Mouse sera were treated the same way human sera were treated. Plates were washed with PBS/0.05% Tween 20 and incubated with horseradish peroxide–conjugated (HRP-conjugated) goat anti–human IgG (H + L) (1:2,000 dilution, Jackson ImmunoResearch) for 1 hour at room temperature. After washing, plates were developed with 100 μL of 3,3′-5,5′ tetramethylbenzidine substrate (Thermo Fisher Scientific) for 90 seconds and fixed with 50 μL of 2N H_2_SO_4_. Plates were read at 450 nm using a Synergy H1 microplate reader (BioTek). Healthy control samples from different days of collection were run on the same plate. All plates were run with the same positive control sample (a healthy donor vaccinated with 3 doses of mRNA vaccine). End point titers were calculated using the average optical density as a cutoff. A specific well was considered positive if optical density signal was 2 times higher than average optical density of blank wells.

### Focus reduction neutralization test.

Serial dilutions of IG products or sera were incubated with 1 × 10^2^ FFU of different strains of SARS-CoV-2 for 1 hour at 37°C. Antibody-virus complexes were added to Vero-TMPRSS2 cell monolayers in 96-well plates and incubated at 37°C for 1 hour. Subsequently, cells were overlaid with 1% (w/v) methylcellulose in MEM supplemented with 2% FBS. Plates were harvested 30 hours (WA1/2020 and Delta) or 68 hours (Omicron BA.1, BQ.1.1, and XBB.1.5) later by removing overlays and fixed with 4% paraformaldehyde (PFA) in PBS for 20 minutes at room temperature. Plates were washed and sequentially incubated with an oligoclonal pool of SARS2-2, SARS2-11, SARS2-16, SARS2-31, SARS2-38, SARS2-57, and SARS2-71 (55, 56) anti-spike antibodies and HRP-conjugated goat anti–mouse IgG (Sigma-Aldrich) in PBS supplemented with 0.1% saponin and 0.1% BSA. SARS-CoV-2–infected cell foci were visualized using TrueBlue peroxidase substrate (KPL) and quantitated on an ImmunoSpot microanalyzer (Cellular Technologies).

### Mouse experiments.

For challenge studies, 7- to 8-week-old female K18-hACE2–transgenic mice were administered (i.p.) PBS or 600 mg/kg IVIG collected before the emergence of COVID-19 or from November 2021 to November 2022. Twenty-four hours later, mice were challenged with 1 × 10^4^ FFU of WA1/2020 D614G or XBB.1.5 in 50 μL by intranasal administration. Daily weights were recorded, and lungs were collected 6 days after infection for virological analysis. In some experiments, 9-week-old male C57BL/6J and FcγR I/III/IV–KO mice were administered 500 μL (50 mg) of IVIG collected from November 2021 to November 2022, 1 day before challenge with 50 μL of 4 × 10^5^ FFU of XBB.1.5 variant by intranasal administration. Lungs were collected 2 days after inoculation for virological analysis.

### Measurement of viral burden.

Lungs were weighed and homogenized with zirconia beads in a MagNA Lyser instrument (Roche Life Science) in 1 mL of DMEM supplemented with 2% heat-inactivated FBS. Tissue homogenates were clarified by centrifugation at 10,000*g* for 5 minutes and stored at −80°C. RNA was extracted using the MagMax mirVana Total RNA isolation kit (Thermo Fisher Scientific) on the Kingfisher Flex extraction robot (Thermo Fisher Scientific). RNA was reverse transcribed and amplified using the TaqMan RNA-to-CT 1-Step Kit (Thermo Fisher Scientific) as described previously (57). Reverse transcription was carried out at 48°C for 15 minutes followed by 2 minutes at 95°C. Amplification was accomplished over 50 cycles consisting of 95°C for 15 seconds and 60°C for 1 minute. Copies of SARS-CoV-2 *N* gene RNA in samples were determined using a published assay (57).

### Viral plaque assay.

Vero-TMPRSS2-hACE2 cells were seeded at a density of 1 × 10^5^ cells per well in 24-well tissue culture plates. The next day, medium was removed and replaced with 200 μL of clarified lung homogenate that was diluted serially in DMEM supplemented with 2% FBS. One hour later, 1 mL of methylcellulose overlay was added. Plates were incubated for 96 hours and then fixed with 4% PFA (final concentration) in PBS for 20 minutes. Plates were stained with 0.05% (w/v) crystal violet in 20% methanol and washed twice with distilled, deionized water.

### Cytokine and chemokine measurements.

Clarified lung homogenates were incubated with Triton X-100 (1% final concentration) for 1 hour at room temperature to inactivate SARS-CoV-2. Homogenates were analyzed for cytokines and chemokines by Eve Technologies Corporation using their Mouse Cytokine Array/Chemokine Array 31-Plex (MD31) platform.

### Statistics.

Statistical significance was assigned when *P* values were less than 0.05 using Prism version 9 (GraphPad). Statistical analysis was determined by 1-way ANOVA with Dunnett’s post hoc test, 2-way ANOVA with Tukey’s post hoc test, paired *t* tests, or Kruskal-Wallis with Dunn’s post hoc test. Tests, number of animals (*n*), mean values, and comparison groups are indicated in the Figure legends.

### Study approval.

The study was approved by the Institutional Review Board of Washington University School of Medicine (approval no. 202104138). All patients signed informed consent. Animal studies were performed in accordance with the NIH *Guide for the Care and Use of Laboratory Animals* (National Academies Press, 2011). The protocols were approved by the Institutional Animal Care and Use Committee at the Washington University School of Medicine (assurance no. A3381-01).

### Material availability.

All requests for resources and reagents should be directed to the corresponding author. This includes viruses, primer-probe sets, and mice. All reagents will be made available on request after completion of a Materials Transfer Agreement.

### Data and code availability.

All data supporting the findings of this study are available within the paper, in the supplemental Supporting Data Values file, and are available from the corresponding author upon request. This paper does not include original code. Any additional information required to reanalyze the data reported in this paper is available from the lead contact upon request.

## Author contributions

OZ designed the study, wrote the study protocol, processed IG products samples, performed SARS-CoV-2 spike protein ELISA experiments, analyzed the data, and supervised the project. AMAD enrolled subjects, collected demographic and clinical data, processed IG products, performed SARS-CoV-2 spike protein ELISA experiments, and analyzed data. CYL, BY, SRM, LAV, and REC designed and performed neutralization experiments and analyzed data. BY, SRM, SMS, and PD preformed in vivo challenge experiments. HJW, TBD, ALK, and ZR provided patient care. TLM wrote the study protocol, managed institutional review board compliance, enrolled individuals, and processed samples. JMM, CCO, and CJR collected demographic and clinical data, enrolled individuals, and processed samples. HGDA processed IG products and performed SARS-CoV-2 spike protein ELISA experiments. LJA and DHF generated crucial reagents. SR planned experiments and analyzed data. AHE and JST contributed samples from the healthy donor cohort. JAO and RMP wrote and maintained the institutional review board protocol, recruited and phlebotomized participants, and coordinated sample collection of healthy donors. PLK contributed to supervision of the project. MSD planned experiments and analyzed data. OZ and MSD wrote the initial draft with detailed comments from AMAD. All other authors provided editorial comments after the first draft.

## Supplementary Material

Supplemental data

Supporting data values

## Version 1 01/04/2024

In-Press Preview

## Version 2 02/08/2024

Electronic publication
