# Supplementary material for: Immunoglobulin replacement products protect against SARS-CoV-2 infection in vivo despite poor neutralizing activity
Source: JCI Insight. 2024 Feb 8;9(3):e176359. doi: 10.1172/jci.insight.176359 (PMC10967375; doi:10.1172/jci.insight.176359)
Supplement: Supplemental data [file jciinsight-9-176359-s234.pdf]

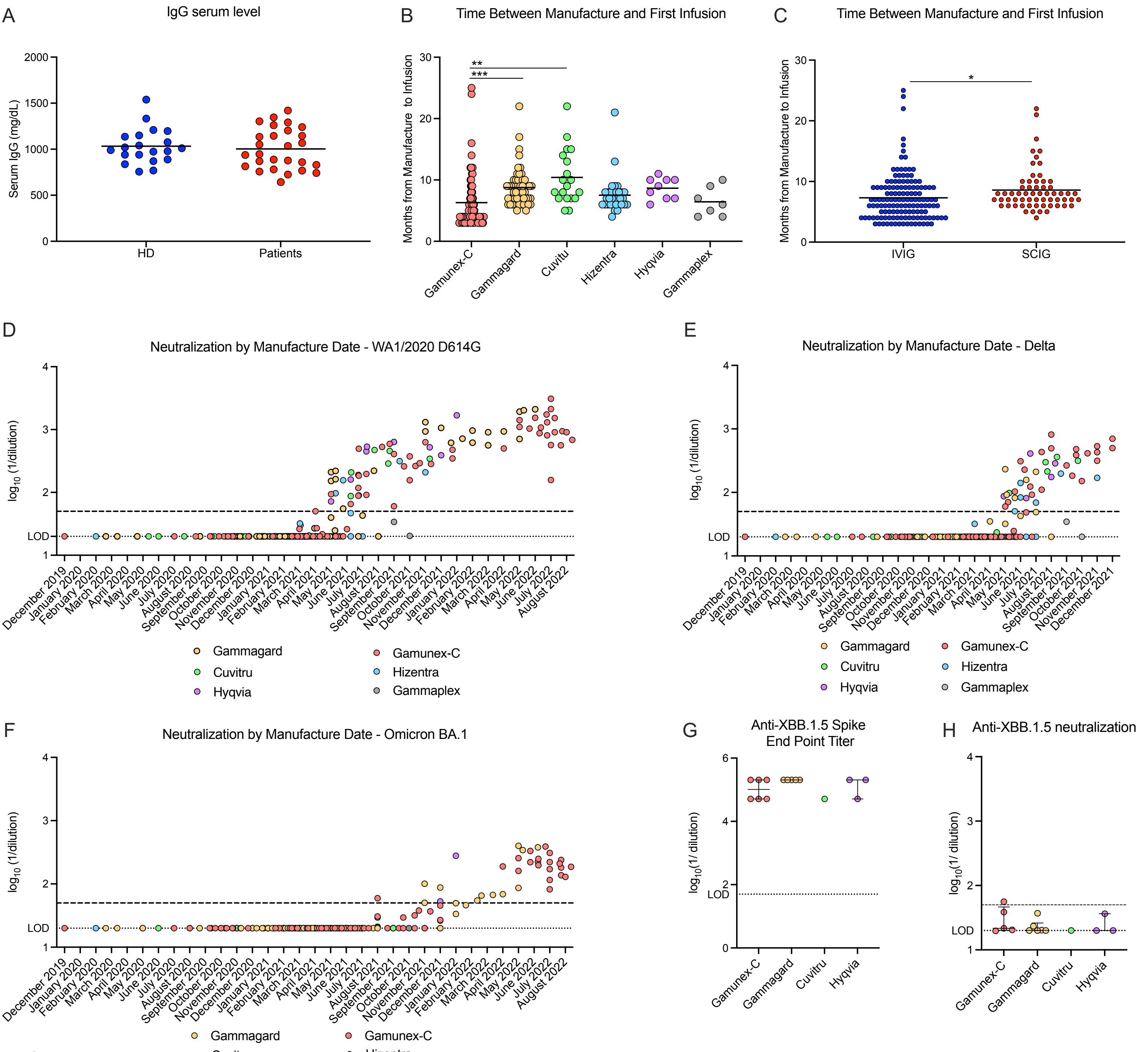
A
IgG serum level
Serum IgG (mg/dL)
HD
Patients
B
Time Between Manufacture and First Infusion
Months from Manufacture to Infusion
**
***
Gamunex-C
Gammagard
Cuvitu
Hizentra
Hyqvia
Gammaplex
C
Time Between Manufacture and First Infusion
Months from Manufacture to Infusion
*
IVIG
SCIG
D
Neutralization by Manufacture Date - WA1/2020 D614G
log10 (1/dilution)
LOD
Gammagard
Cuvitru
Hyqvia
Gamunex-C
Hizentra
Gammaplex
E
Neutralization by Manufacture Date - Delta
log10 (1/dilution)
LOD
Gammagard
Cuvitru
Hyqvia
Gamunex-C
Hizentra
Gammaplex
F
Neutralization by Manufacture Date - Omicron BA.1
log10 (1/dilution)
LOD
Gammagard
Cuvitru
Hyqvia
Gamunex-C
Hizentra
Gammaplex
G
Anti-XBB.1.5 Spike
End Point Titer
log10(1/ dilution)
LOD
Gamunex-C
Gammagard
Cuvitru
Hyqvia
H
Anti-XBB.1.5 neutralization
log10(1/ dilution)
LOD
Gamunex-C
Gammagard
Cuvitru
Hyqvia

S1

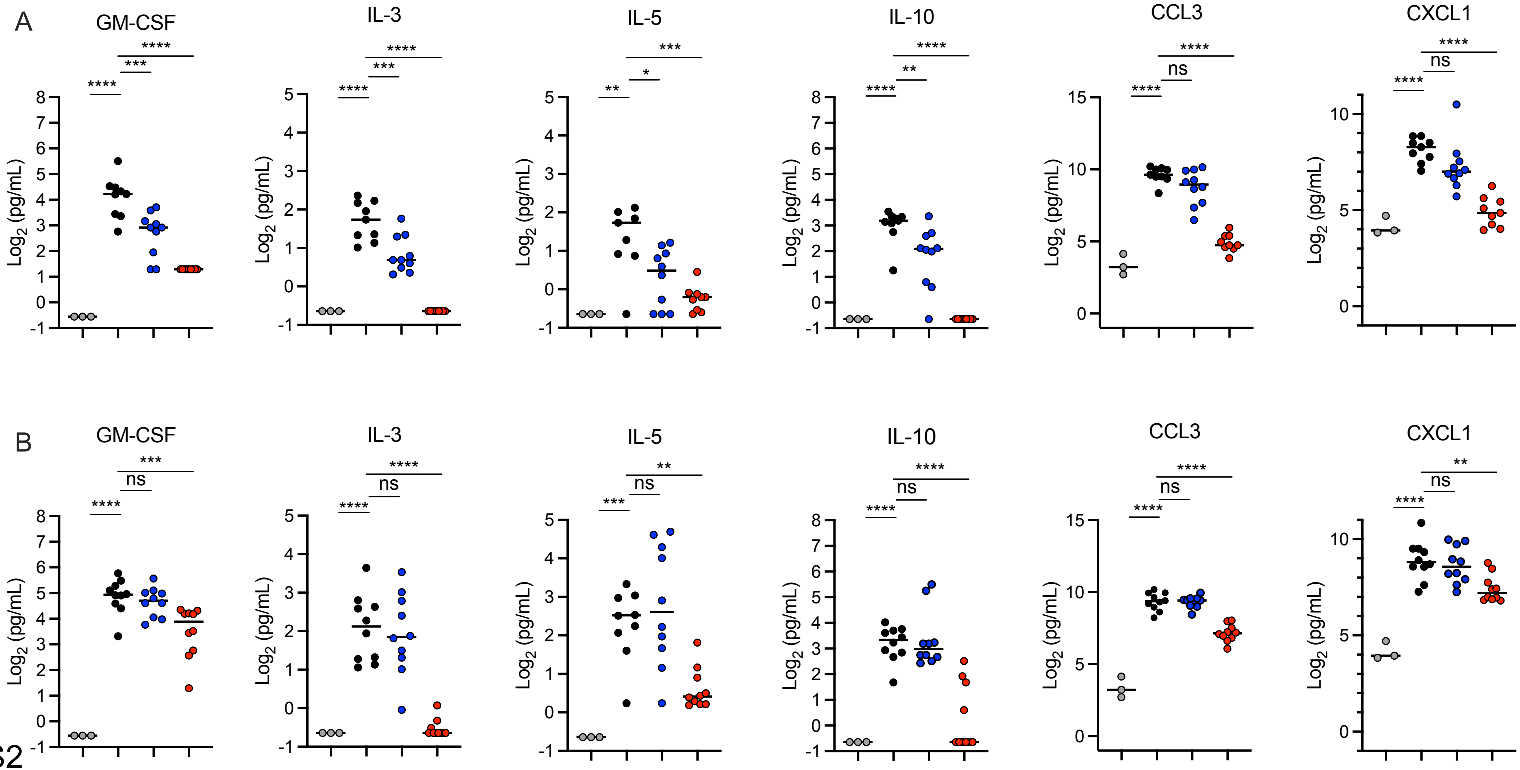
A
GM-CSF
IL-3
IL-5
IL-10
CCL3
CXCL1
B
GM-CSF
IL-3
IL-5
IL-10
CCL3
CXCL1
Log2 (pg/mL)
ns

S2

Table S1. Anti-spike antibody titer and SARS-CoV-2 neutralization activity in 198 IVIG and SCIG lots.

IVIG product	Lot number	Expiration date (refrigerated)	Manufacture Date	First Infusion Month	Anti-Wuhan-1 spike antibody end point titers	WA1/2020 Neutralization	Delta Neutralization	Omicron BA.1 Neutralization	Omicron BQ.1.1 Neutralization	Anti-XBB.1.5 spike antibody end point titers	XBB.1.5 Neutralization
CUVITRU	LE13W047AB	May-23	May-20	August-21	50	20.00	20	20	--	--	--
CUVITRU	LE13W055AC	June-23	June-20	March-22	3200	20.00	20	20	--	--	--
CUVITRU	LE13W070AB	August-23	August-20	September-21	200	20.00	20	20	--	--	--
CUVITRU	LE13W097AC	October-23	October-20	August-21	200	20.00	20	20	--	--	--
CUVITRU	LE13W103AB	November-23	November-20	October-21	200	20.00	20	20	--	--	--
CUVITRU	LE13X057AC	November-23	November-20	April-22	200	20.00	20	20	--	--	--
CUVITRU	LE13W109AB	November-23	November-20	January-22	200	20.00	20	20	--	--	--
CUVITRU	LE13W109AC	November-23	November-20	February-22	3200	20.00	20	20	--	--	--
CUVITRU	LE13X012AB	February-24	February-21	September-21	800	20.00	20	20	--	--	--
CUVITRU	LE13X020AB	February-24	February-21	October-21	800	20.00	20	20	--	--	--
CUVITRU	LE13X032AB	March-24	March-21	August-21	800	20.00	20	20	--	--	--
CUVITRU	LE13X039AB	April-24	April-21	August-21	800	20.00	20	20	--	--	--
CUVITRU	LE13X048AB	May-24	May-21	January-22	3200	20.02	23.5	20	--	--	--
CUVITRU	LE13X059AB	June-24	June-21	March-22	3200	87.33	97.85	20	--	--	--
CUVITRU	LE13X073AB	July-24	July-21	February-22	51200	207.90	213.8	20	--	--	--
CUVITRU	LE13X084AB	August-24	August-21	March-22	12800	473.40	298.6	29.41	--	--	--
CUVITRU	LE13X087AC	August-24	August-21	April-22	51200	455.60	361.1	20.68	--	--	--
CUVITRU	LE13X088AB	September-24	September-21	March-22	51200	287.90	315.4	20	--	--	--
CUVITRU	LE13X133AB	December-24	December-21	October-22	12800	342.00	--	20	20	51200	20.00

IVIG product	Lot number	Expiration date (refrigerated)	Manufacture Date	First Infusion Month	Anti-Wuhan-1 spike antibody end point titers	Washington Neutralization	Delta Neutralization	Omicron Neutralization	BQ.1.1	Anti-XBB.1.5 spike antibody end point titers	XBB.1.5 Neutralization
Gammagard	LE08W010AD	March-22	March-20	August-21	50	20.00	20	20	--	--	--
Gammagard	LE08W010AE	March-22	March-20	January-22	50	20.00	20	20	--	--	--
Gammagard	LE08W016AB	May-22	May-20	August-21	800	20.00	20	20	--	--	--
Gammagard	LE08W026AD	September-22	September-20	August-21	800	20.00	20	20	--	--	--
Gammagard	LE08W033AC	November-22	November-20	September-21	3200	20.00	20	20	--	--	--
Gammagard	LE08W036AD	November-22	November-20	September-21	200	20.00	20	20	--	--	--
Gammagard	LE08W037AE	November-22	November-20	August-21	3200	20.00	20	20	--	--	--
Gammagard	LE12X024AC	December-23	December-20	January-22	200	20.00	20	20	--	--	--
Gammagard	LE08W038AC	December-22	December-20	August-21	200	20.00	20	20	--	--	--
Gammagard	LE08W039AC	December-22	December-20	August-21	3200	20.00	20	20	--	--	--
Gammagard	C21G010AAA	January-24	January-21	August-21	800	20.00	20	20	--	--	--
Gammagard	LE08X001AC	January-23	January-21	October-21	200	20.00	20	20	--	--	--
Gammagard	LE08X002AC	January-23	January-21	October-21	200	20.00	20	20	--	--	--
Gammagard	LE12X021AB	January-24	January-21	August-21	200	20.00	20	20	--	--	--
Gammagard	le08x003ac	February-23	February-21	November-21	200	20.00	20	20	--	--	--
Gammagard	LE12X034AB	February-24	February-21	August-21	200	20.00	20	20	--	--	--
Gammagard	LE08X004AC	February-23	February-21	December-21	800	20.00	20	20	--	--	--
Gammagard	LE08X005AE	February-23	February-21	November-21	200	20.00	20	20	--	--	--
Gammagard	LE08X005AJ	February-23	February-21	November-21	200	20.00	20	20	--	--	--
Gammagard	LE12X043AB	February-24	February-21	September-21	200	20.00	20	20	--	--	--
Gammagard	C21G029AAA	May-24	March-21	October-21	800	20.00	20	20	--	--	--
Gammagard	LE08X006AC	March-23	March-21	December-21	800	20.00	20	20	--	--	--
Gammagard	LE12X072AB	March-24	March-21	September-21	800	20.00	20	20	--	--	--
Gammagard	LE12X073AB	March-24	March-21	November-21	800	20.00	20	20	--	--	--
Gammagard	LE08X008AC	March-23	March-21	January-22	200	20.00	20	20	--	--	--
Gammagard	C21G036AAA	March-24	March-21	September-21	800	20.00	20	20	--	--	--
Gammagard	LE12X087AB	March-24	March-21	December-21	3200	20.52	20	20	--	--	--
Gammagard	LE08X010AE	April-23	April-21	February-22	200	20.00	20	20	--	--	--
Gammagard	LE12X110AB	April-24	April-21	November-21	3200	26.87	35.05	20	--	--	--
Gammagard	LE08X012AC	May-23	May-21	January-22	800	20.00	20	20	--	--	--
Gammagard	C21G044AAA	May-24	May-21	October-21	3200	24.74	20	20	--	--	--
Gammagard	LE12X136AB	May-24	May-21	November-21	12800	39.77	32.03	20	--	--	--
Gammagard	LE12X141AB	May-24	May-21	January-22	12800	152.40	232.3	20	--	--	--
Gammagard	LE08X013AG	May-23	May-21	December-21	51200	210.00	92	20	--	--	--
Gammagard	C21G049AAA	June-24	June-21	December-21	12800	55.14	42.6	20	--	--	--
Gammagard	LE12X157AB	June-24	June-21	December-21	12800	162.70	157.7	20	--	--	--
Gammagard	LE12X160AB	June-24	June-21	December-21	800	20.00	20	20	--	--	--
Gammagard	LE12X165AB	June-24	June-21	February-22	51200	220.00	82	20	--	--	--
Gammagard	LE12X171AB	July-24	July-21	January-22	12800	220.90	211.2	20	--	--	--
Gammagard	C21G067AAA	July-24	July-21	February-22	51200	42.81	49.02	20	--	--	--
Gammagard	LE12X187AB	August-24	August-21	February-22	12800	20.00	20	20	--	--	--
Gammagard	LE08X026AC	November-23	November-21	October-22	204800	1309.00	--	100.6	25.79	204800	23.35
Gammagard	LE08X027AC	November-23	November-21	November-22	204800	934.20	--	50.31	25.15	204800	20.00
Gammagard	LE08X029AC	December-23	December-21	October-22	12800	1063.00	--	87.75	20	204800	20.21
Gammagard	BE08C003AH	January-24	January-22	October-22	51200	615.20	--	33.45	21.28	--	--
Gammagard	BE08C003AJ	January-24	January-22	October-22	204800	715.10	--	49.68	20	204800	20.00
Gammagard	BE08C005AC	February-24	February-22	October-22	204800	961.60	--	54.77	20	204800	20.00
Gammagard	BE08C006AC	February-24	February-22	November-22	204800	618.80	--	46.14	20	--	--
Gammagard	BE08C007AC	March-24	March-22	November-22	204800	904.60	--	65.4	21.14	--	--
Gammagard	C22G049AAA	March-25	March-22	October-22	51200	562.90	--	67.02	20	--	--

Gammagard	BE08C010AC	April-24	April-22	November-22	204800	933.40	--	68.86	23.91	--	--
Gammagard	BE12CA96AB	May-25	May-22	October-22	51200	706.20	--	86.57	23	--	--
Gammagard	BE12C105AB	May-25	May-22	November-22	204800	1928.00	--	400.2	86.6	--	--
Gammagard	BE12C114AB	June-25	June-22	November-22	204800	2105.00	--	342.9	82.34	--	--
Gammagard	BE12CC27AB	June-25	June-22	November-22	51200	2027.00	--	378	30.68	204800	36.99

IVIG product	Lot number	Expiration date (refrigerated)	Manufacture Date	First Infusion Month	Anti-Wuhan-1 spike antibody end point titers	Washington Neutralization	Delta Neutralization	Omicron Neutralization	BQ.1.1	Anti-XBB.1.5 spike antibody end point titers	XBB.1.5 Neutralization
Gammaplex	GSB21226R	January-24	January-21	October-21	200	20.00	20	20	--	--	--
Gammaplex	65B21127	April-24	April-21	August-21	800	20.00	20	20	--	--	--
Gammaplex	GSB21134	April-24	April-21	September-21	200	20.00	20	20	--	--	--
Gammaplex	GSB21180	May-24	May-21	November-21	800	20.00	20	20	--	--	--
Gammaplex	GSC21216	May-24	May-21	January-22	3200	20.00	20	20	--	--	--
Gammaplex	GSB21274	September-24	September-21	December-21	3200	33.97	34.51	20	--	--	--
Gammaplex	GSB21297	October-24	October-21	January-22	3200	20.31	20	20	--	--	--

IVIG product	Lot number	Expiration date (refrigerated)	Manufacture Date	First Infusion Month	Anti-Wuhan-1 spike antibody end point titers	Washington Neutralization	Delta Neutralization	Omicron Neutralization	BQ.1.1	Anti-XBB.1.5 spike antibody end point titers	XBB.1.5 Neutralization
Gamunex-C	B3GJD00383	December-22	December-19	November-21	50	20.00	20	20	--	--	--
Gamunex-C	B2GJE00483	July-23	July-20	September-21	50	20.00	20	20	--	--	--
Gamunex-C	B3GLE01322	August-23	August-20	August-21	200	20.00	20	20	--	--	--
Gamunex-C	B3GJE00633	September-23	September-20	August-21	800	20.00	20	20	--	--	--
Gamunex-C	B1GKE00453	October-23	October-20	August-21	800	20.00	20	20	--	--	--
Gamunex-C	B8GJE00733	October-23	October-20	August-21	800	20.00	20	20	--	--	--
Gamunex-C	B8GJE00743	October-23	October-20	September-21	800	20.00	20	20	--	--	--
Gamunex-C	A3GJE00693	October-23	October-20	August-21	200	20.00	20	20	--	--	--
Gamunex-C	B26HE00113	October-23	October-20	November-22	50	--	--	--	--	--	--
Gamunex-C	A3GJE00753	October-23	October-20	September-21	200	20.00	20	20	--	--	--
Gamunex-C	B1GJE00803	October-23	November-20	February-22	200	20.00	20	20	--	--	--
Gamunex-C	B8GMF00173	January-24	January-21	November-21	800	20.00	20	20	--	--	--
Gamunex-C	A1GKF00072	February-24	February-21	August-21	200	20.00	20	20	--	--	--
Gamunex-C	B2GKF00113	February-24	February-21	August-21	200	20.00	20	20	--	--	--
Gamunex-C	B3GJF00163	March-24	March-21	September-21	800	20.00	20	20	--	--	--
Gamunex-C	B2GJF00213	March-24	March-21	October-21	800	20.00	20	20	--	--	--
Gamunex-C	A4GKF00112	March-24	March-21	September-21	800	20.00	20	20	--	--	--
Gamunex-C	A4GKF00132	March-24	March-21	October-21	800	20.00	20	20	--	--	--
Gamunex-C	B3GKF00183	March-24	March-21	August-21	3200	20.00	20	20	--	--	--
Gamunex-C	B1GJF00323	March-24	March-21	December-21	800	26.30	20	20	--	--	--
Gamunex-C	B8GKF00243	April-24	April-21	August-21	3200	20.00	20	20	--	--	--
Gamunex-C	B8GMF00363	April-24	April-21	August-21	800	50.12	--	--	--	--	--
Gamunex-C	A4GMF00383	April-24	April-21	August-21	3200	20.00	20	20	--	--	--
Gamunex-C	B8GKF00233	April-24	April-21	September-21	3200	20.00	20	20	--	--	--
Gamunex-C	B8GMF00403	April-24	April-21	August-21	12800	27.00	20	20	--	--	--
Gamunex-C	B3GJF00453	April-24	May-21	January-22	3200	20.00	20	20	--	--	--
Gamunex-C	B8GJF00463	May-24	May-21	December-21	3200	21.63	24.71	20	--	--	--
Gamunex-C	A4GLF00642	May-24	May-21	August-21	800	20.00	20	20	--	--	--
Gamunex-C	B1GKF00283	May-24	May-21	November-21	3200	20.00	70.11	20	--	--	--
Gamunex-C	B2GMF00453	May-24	May-21	September-21	3200	20.00	20	20	--	--	--
Gamunex-C	B8GMF00503	May-24	May-21	August-21	51200	93.69	60.01	20	--	--	--
Gamunex-C	A1GKF00312	June-24	June-21	November-21	12800	117.60	102.9	20.00	--	--	--
Gamunex-C	B8GLF00653	May-24	June-21	August-21	3200	65.41	48.63	20	--	--	--
Gamunex-C	A4GLF00663	May-24	June-21	August-21	3200	20.00	20	20	--	--	--
Gamunex-C	A1GKF00272	June-24	June-21	December-21	12800	88.47	91.95	20	--	--	--
Gamunex-C	B1GLF00683	June-24	June-21	August-21	12800	26.00	20	20	--	--	--
Gamunex-C	B8GKF00343	June-24	June-21	December-21	12800	190.10	309.2	20	--	--	--
Gamunex-C	B2GJF00033	January-24	June-21	September-21	800	20.00	20	20	--	--	--
Gamunex-C	A4GLF00792	July-24	July-21	August-21	12800	196.70	228.2	20	--	--	--
Gamunex-C	A1GLF00802	July-24	July-21	October-21	51200	495.60	432	20	--	--	--
Gamunex-C	B8GKF00353	July-24	July-21	January-22	51200	184.40	123.6	20	--	--	--
Gamunex-C	B1GJF00713	July-24	July-21	April-22	12800	91.33	110.2	20	--	--	--
Gamunex-C	A4GLF00922	July-24	August-21	October-21	51200	528.40	495.3	30.62	--	--	--
Gamunex-C	A1GLF01042	August-24	August-21	November-21	51200	593.20	814.2	59.52	--	--	--
Gamunex-C	B4GLF00923	August-24	September-21	December-21	12800	60.24	265.9	20	--	--	--
Gamunex-C	A01F000572	September-24	September-21	January-22	204800	407.00	183	20	--	--	--
Gamunex-C	A01F002332	October-24	October-21	December-21	51200	374.20	386.5	20	--	--	--
Gamunex-C	A04F005362	October-24	October-21	March-22	51200	263.10	479.5	29.46	--	--	--
Gamunex-C	B08F008573	October-24	October-21	February-22	51200	259.80	413.3	31.78	--	--	--
Gamunex-C	B01F009943	October-24	October-21	April-22	12800	156.30	151.2	20	--	--	--
Gamunex-C	A01F009322	November-24	November-21	February-22	51200	291.50	429.9	38.01	--	--	--
Gamunex-C	B01F011803	November-24	November-21	April-22	12800	282.80	315	20	--	--	--
Gamunex-C	B08F012473	November-24	November-21	February-22	12800	627.90	534.5	36.91	--	--	--
Gamunex-C	A01F018912	December-24	December-21	March-22	51200	475.70	699.5	45.47	--	--	--
Gamunex-C	B08G000473	December-24	December-21	April-22	51200	346.40	498.5	26.94	--	--	--
Gamunex-c	B02G074903	April-25	April-22	November-22	51200	499.10	--	189.3	56.32	--	--

Gamunex-c	B08G040013	May-25	May-22	October-22	204800	1102.00	--	160.4	21.44	204800	20.00
Gamunex-c	A01G041723	May-25	May-22	October-22	204800	1411.00	--	254.9	30.84	51200	21.64
Gamunex-c	A01G037042	June-25	June-22	October-22	204800	1537.00	--	389.2	51.42	204800	38.64
Gamunex-c	B036048513	June-25	June-22	November-22	51200	1735.00	--	331.6	59.07	--	--
Gamunex-c	B026048603	June-25	June-22	October-22	204800	1079.00	--	223.7	36.96	--	--
Gamunex-c	B026038632	June-25	June-22	October-22	204800	1034.00	--	220.9	33.82	--	--
Gamunex-c	B016039282	July-25	July-22	October-22	204800	1523.00	--	309.3	54.84	51200	55.81
Gamunex-c	R026055743	July-25	July-22	October-22	204800	875.00	--	181.5	39.39	51200	20.39
Gamunex-c	B016058583	July-25	July-22	November-22	204800	907.50	--	239.9	71.79	--	--
Gamunex-c	B016058243	July-25	July-22	November-22	204800	857.90	--	170.8	27.38	--	--
Gamunex-c	B03G059323	July-25	July-22	November-22	51200	567.80	--	137.1	26.55	--	--
Gamunex-c	A016039862	July-25	July-22	November-22	51200	3093.00	--	249.4	44.79	--	--
Gamunex-c	B016061903	July-25	July-22	November-22	204800	808.00	--	221.6	31.59	--	--
Gamunex-c	A046061913	July-25	July-22	November-22	51200	942.60	--	82.18	20	--	--
Gamunex-c	B03G063163	July-25	July-22	November-22	51200	1303.00	--	197.1	27.32	--	--
Gamunex-c	A046063263	July-25	July-22	November-22	51200	2121.00	--	115.4	20.43	--	--
Gamunex-c	B01G067063	August-25	August-22	November-22	204800	686.50	--	209.1	24.15	--	--
Gamunex-c	B016045372	August-25	August-22	November-22	204800	904.60	--	186.4	32.86	--	--
Gamunex-c	B01G048792	August-25	August-22	November-22	51200	561.20	--	129.2	31.14	--	--

IVIG product	Lot number	Expiration date (refrigerated)	Manufacture Date	First Infusion Month	Anti-Wuhan-1 spike antibody end point titers	Washington Neutralization	Delta Neutralization	Omicron Neutralization	BQ.1.1	Anti-XBB.1.5 spike antibody end point titers	XBB.1.5 Neutralization
Hizentra	P100299150	August-22	February-20	October-21	50	20.00	20	20	--	--	--
Hizentra	P100283113	May-23	November-20	November-21	50	20.00	20	20	--	--	--
Hizentra	p100295167	June-23	December-20	August-21	800	20.00	20	20	--	--	--
Hizentra	p100298818	July-23	January-21	September-21	800	20.00	20	20	--	--	--
Hizentra	P100301497	July-23	January-21	August-21	800	20.00	20	20	--	--	--
Hizentra	P100303965	July-23	January-21	August-21	800	20.00	20	20	--	--	--
Hizentra	p100305419	August-23	February-21	August-21	800	20.00	20	20	--	--	--
Hizentra	p100308202	August-23	February-21	September-21	800	20.00	20	20	--	--	--
Hizentra	P100308204	August-23	February-21	August-21	200	20.00	20	20	--	--	--
Hizentra	P100313109	August-23	February-21	August-21	200	20.00	20	20	--	--	--
Hizentra	P100317961	September-23	March-21	August-21	800	20.00	20	20	--	--	--
Hizentra	P100317965	September-23	March-21	August-21	800	20.00	20	20	--	--	--
Hizentra	P100317968	September-23	March-21	September-21	800	20.00	20	20	--	--	--
Hizentra	P100320293	September-23	March-21	November-21	800	20.00	20	20	--	--	--
Hizentra	P100320296	September-23	March-21	August-21	3200	30.00	32	20	--	--	--
Hizentra	P100320297	September-23	March-21	October-21	200	20.00	20	20	--	--	--
Hizentra	P100325992	September-23	March-21	August-21	800	20.00	20	20	--	--	--
Hizentra	P100325987	September-23	March-21	October-21	800	20.00	20	20	--	--	--
Hizentra	P100325989	September-23	March-21	November-21	800	32.09	--	--	--	--	--
Hizentra	P100325993	September-23	March-21	September-21	3200	20.00	20	20	--	--	--
Hizentra	P100325990	October-23	April-21	September-21	800	20.00	20	20	--	--	--
Hizentra	P100328020	October-23	April-21	December-21	800	20.00	20	20	--	--	--
Hizentra	P100329758	October-23	April-21	September-21	200	20.00	20	20	--	--	--
Hizentra	P100334886	October-23	April-21	October-21	3200	20.00	20	20	--	--	--
Hizentra	P100340458	November-23	May-21	December-21	200	20.00	20	20	--	--	--
HIzentra	P100345824	December-23	June-21	November-21	3200	46.76	50.46	20	--	--	--
HIzentra	P100348234	December-23	June-21	February-22	12800	97.10	83.4	20	--	--	--
Hizentra	P100354761	December-23	June-21	January-22	12800	155.90	140.8	20	--	--	--
Hizentra	P100354768	December-23	June-21	February-22	3200	20.00	20	20	--	--	--
Hizentra	P100359895	January-24	July-21	March-22	800	20.00	20	20	--	--	--
Hizentra	P100359898	January-24	July-21	March-22	12800	89.79	69.12	20	--	--	--
Hizentra	P100371306	March-24	September-21	February-22	12800	314.30	198.2	20	--	--	--
Hizentra	P100386620	May-24	November-21	March-22	51200	209.10	169.4	20	--	--	--

IVIG product	Lot number	Expiration date (refrigerated)	Manufacture Date	First Infusion Month	Anti-Wuhan-1 spike antibody end point titers	Washington Neutralization	Delta Neutralization	Omicron Neutralization	BQ.1.1	Anti-XBB.1.5 spike antibody end point titers	XBB.1.5 Neutralization
Hyqvia	LE16X003AA	November-23	January-21	September-21	50	20.00	20	20	--	--	--
Hyqvia	LE16X129AA	March-23	May-21	March-22	12800	71.79	87.01	20	--	--	--
Hyqvia	LE16X156AA	March-23	June-21	December-21	12800	160.10	81.33	20	--	--	--
Hyqvia	LE16X184AA	July-24	July-21	March-22	51200	450.60	410.1	20	--	--	--
Hyqvia	LE16X192AA	July-24	August-21	February-22	51200	533.00	175	21	--	--	--
Hyqvia	LE16X217AA	July-24	September-21	March-22	51200	634.00	287	20	--	--	--
Hyqvia	BE16C005AB	December-24	December-21	October-22	12800	390.80	--	26.35	20	51200	20.00
Hyqvia	BE16C068AA	December-24	December-21	October-22	12800	522.90	--	52.83	20	204800	20.00
Hyqvia	BE16C097AA	January-25	January-22	November-22	51200	1682.00	--	278.8	48.69	204800	36.36
